# *catena*-Poly[[aqua­(ethyl anilinophospho­nato-κ*O*)sodium(I)]-di-μ-aqua]

**DOI:** 10.1107/S1600536808025233

**Published:** 2008-08-13

**Authors:** Zhiyong Fu, Shuqiong Bai

**Affiliations:** a School of Chemistry and Chemical Engineering, South China University of Technology, Guangzhou, People’s Republic of China

## Abstract

In the title compound, [Na(C_8_H_11_NO_3_P)(H_2_O)_3_]_*n*_, the sodium cation is octa­hedrally coordinated by five water mol­ecules and one O-bonded ethyl anilinophospho­nate anion. Four of the water mol­ecules bridge to adjacent sodium ions, resulting in an infinite chain of edge-sharing NaO_6_ polyhedra. A network of N—H⋯O and O—H⋯O hydrogen bonds helps to stabilize the crystal structure.

## Related literature

For the corresponding zinc complex, see: Fu & Chivers (2005 ▶). For background, see: Cheetham *et al.* (1999 ▶); Andrianov *et al.* (1977 ▶).
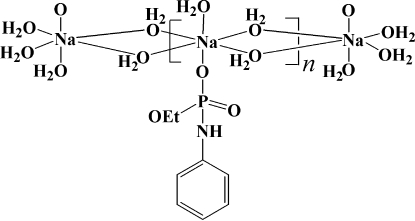


## Experimental

### 

#### Crystal data

[Na(C_8_H_11_NO_3_P)(H_2_O)_3_]
*M* *_r_* = 277.19
Monoclinic, 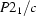

*a* = 17.332 (4) Å
*b* = 5.2591 (11) Å
*c* = 14.009 (3) Å
β = 100.37 (3)°
*V* = 1256.1 (5) Å^3^
*Z* = 4
Mo *K*α radiation
μ = 0.27 mm^−1^
*T* = 173 (2) K
0.20 × 0.12 × 0.10 mm

#### Data collection

Siemens SMART CCD diffractometer
Absorption correction: multi-scan (*SADABS*; Siemens, 1996 ▶) *T*_min_ = 0.961, *T*_max_ = 0.977
3993 measured reflections
2145 independent reflections
1716 reflections with *I* > 2σ(*I*)
*R*_int_ = 0.024

#### Refinement

*R*[*F* ^2^ > 2σ(*F* ^2^)] = 0.032
*wR*(*F* ^2^) = 0.076
*S* = 1.05
2145 reflections
178 parameters
H atoms treated by a mixture of independent and constrained refinement
Δρ_max_ = 0.19 e Å^−3^
Δρ_min_ = −0.25 e Å^−3^

### 

Data collection: *SMART* (Siemens, 1996 ▶); cell refinement: *SAINT* (Siemens, 1994 ▶); data reduction: *SAINT*; program(s) used to solve structure: *SHELXS97* (Sheldrick, 2008 ▶); program(s) used to refine structure: *SHELXL97* (Sheldrick, 2008 ▶); molecular graphics: *SHELXTL* (Sheldrick, 2008 ▶); software used to prepare material for publication: *SHELXTL*.

## Supplementary Material

Crystal structure: contains datablocks global, I. DOI: 10.1107/S1600536808025233/hb2768sup1.cif

Structure factors: contains datablocks I. DOI: 10.1107/S1600536808025233/hb2768Isup2.hkl

Additional supplementary materials:  crystallographic information; 3D view; checkCIF report

## Figures and Tables

**Table 1:** Selected bond lengths (Å)

Na1—O6^i^	2.3818 (16)
Na1—O6	2.4061 (16)
Na1—O4	2.4113 (16)
Na1—O1	2.4543 (15)
Na1—O5	2.5014 (18)
Na1—O5^ii^	2.4902 (17)

Symmetry code: (i) 
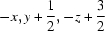
; (ii) 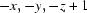.

**Table 2:** Hydrogen-bond geometry (Å, °)

*D*—H⋯*A*	*D*—H	H⋯*A*	*D*⋯*A*	*D*—H⋯*A*
N1—H1*A*⋯O3^ii^	0.86	2.29	3.132 (2)	166
O4—H1⋯O1^iii^	0.81 (3)	2.02 (3)	2.808 (2)	165 (2)
O4—H2⋯O2^i^	0.82 (2)	1.92 (3)	2.693 (2)	159 (2)
O5—H4⋯O1^ii^	0.82 (4)	2.32 (3)	3.116 (2)	162 (3)
O6—H5⋯O4^iv^	0.80 (3)	2.01 (3)	2.797 (2)	171 (3)
O6—H6⋯O2^i^	0.83 (3)	1.96 (3)	2.771 (2)	166 (3)

Symmetry codes: (i) 
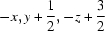
; (ii) 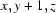; (iii) 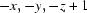; (iv) 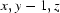.

**Fig. 1.**
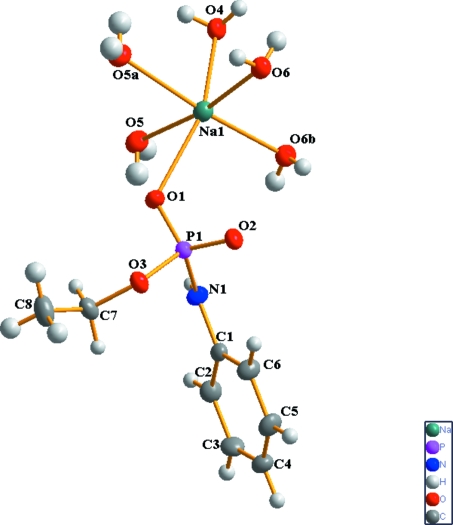
The molecular structure of (I), with 50% probability displacement ellipsoids for non-H atoms. Atoms O5a and O6a are generated by the symmetry operations (-x, -y, 1-z) and (-x, 1/2+y, 3/2-z), respectively.

**Fig. 2.**
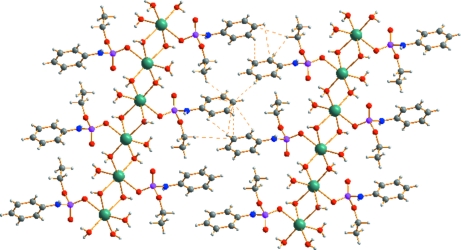
The packing of (I), viewed down the b axis.

## supplementary crystallographic information

### Comment

Metal phosphates have attracted immense interest during the last two decades for their applications as molecular sieves, absorbents and catalysts (Cheetham *et al.*, 1999). However, the crystal structures of their analogous complex metal phosphate oxynitrides are not well characterized (Fu *et al.*, 2005). As part of our investigation of these materials, the title compound, (I), was prepared and characterised.

The asymmetric unit of (I) is composed of three water molecules, one *N*-ethoxyphosphorl-phenyl-amide anion and one sodium cation (Fig. 1). The central Na atom has a slightly distorted octahedral coordination mode with six oxygen atoms around it (Table 1). One belongs the *N*-ethoxyphosphorl-phenyl-amide anion, while all the others are coming from the water molecules. The Na—O bond lengths range from 2.3818 (16) to 2.5014 (18) Å. The phosphorus atom of the ligand adopts a tetrahedral coordination mode and there are three types of P—O bonds and one P—N bond existing in the [PO_3_N] tetrahetra. The shortest bond lengths of 1.4885 (14) Å (P1—O2) refers to the P=O double bond and the P—O bond lengths of 1.6089 (14)Å is attributed to the P-OEt connection. The longer P—O distance is due to the influence of the –OEt group, according to the literature report (Andrianov *et al.*,1977). The P—N bond length is 1.6612 (16) Å. The bond angles of O—P—O and O—P—N range from 103.77 (8)–118.43 (8)° and 105.43 (8)–112.93 (8)°, indicating that the tetrahetron is slightly distorted. The connections between the sodium ions and the water molecules result an infinite chain (Figure 2), with the *N*-ethoxyphosphorl-phenyl-amide anions are appended beside the chains. The phenyl rings have an edge on T shaped stacking geometry and they are overlapped in a parallel displaced mode.  A network of N—H···O and O—H···O hydrogen bonds (Table 2) helps to establish the packing.

### Experimental

A solution of NaOH (3 mmol) and [Et_2_NH_2_][(EtO)PO_2_(C_6_H_5_NH)] (1 mmol) in 10 ml H_2_O was stirred for 21 h at room temperature. Colourless blocks of (I) were obtained after one week.

### Refinement

The H atoms bonded to the O atoms of the water molecules were located in a difference map and refined with distance restraints of O—H = 0.85 (3) Å and isotropic displacement parameters. The other H atoms were positioned geometrically and refined using a riding model approximation, with C—H = 0.93–0.97Å and with *U*_iso_(H)= 1.2U_eq_(C) or 1.5U_eq_(methyl C).

### Crystal data

**Table:**

[Na(C_8_H_11_NO_3_P)(H_2_O)_3_]	*F*_000_ = 584
*M**_r_* = 277.19	*D*_x_ = 1.466 Mg m^−^^3^
Monoclinic, *P*2_1_/*c*	Mo *K*α radiation λ = 0.71073 Å
Hall symbol: -P 2ybc	Cell parameters from 3993 reflections
*a* = 17.332 (4) Å	θ = 4.1–25.0º
*b* = 5.2591 (11) Å	µ = 0.27 mm^−^^1^
*c* = 14.009 (3) Å	*T* = 173 (2) K
β = 100.37 (3)º	Block, colourless
*V* = 1256.1 (5) Å^3^	0.20 × 0.12 × 0.10 mm
*Z* = 4	

### Data collection

**Table:**

Siemens SMART CCD diffractometer	1716 reflections with *I* > 2σ(*I*)
Radiation source: fine-focus sealed tube	*R*_int_ = 0.024
Monochromator: graphite	θ_max_ = 25.0º
ω scans	θ_min_ = 4.1º
Absorption correction: multi-scan(SADABS; Siemens, 1996)	*h* = −20→20
*T*_min_ = 0.961, *T*_max_ = 0.977	*k* = −6→6
3993 measured reflections	*l* = −16→16
2145 independent reflections	

### Refinement

**Table:**

Refinement on *F*^2^	Secondary atom site location: difference Fourier map
Least-squares matrix: full	Hydrogen site location: difmap and geom
*R*[*F*^2^ > 2σ(*F*^2^)] = 0.032	H atoms treated by a mixture of independent and constrained refinement
*wR*(*F*^2^) = 0.076	*w* = 1/[σ^2^(*F*_o_^2^) + (0.0341*P*)^2^ + 0.4043*P*] where *P* = (*F*_o_^2^ + 2*F*_c_^2^)/3
*S* = 1.05	(Δ/σ)_max_ < 0.001
2145 reflections	Δρ_max_ = 0.19 e Å^−^^3^
178 parameters	Δρ_min_ = −0.25 e Å^−^^3^
Primary atom site location: structure-invariant direct methods	Extinction correction: none

### Special details

**Table:**

Geometry. All e.s.d.'s (except the e.s.d. in the dihedral angle between two l.s. planes) are estimated using the full covariance matrix. The cell e.s.d.'s are taken into account individually in the estimation of e.s.d.'s in distances, angles and torsion angles; correlations between e.s.d.'s in cell parameters are only used when they are defined by crystal symmetry. An approximate (isotropic) treatment of cell e.s.d.'s is used for estimating e.s.d.'s involving l.s. planes.
Refinement. Refinement of *F*^2^ against ALL reflections. The weighted *R*-factor *wR* and goodness of fit *S* are based on *F*^2^, conventional *R*-factors *R* are based on *F*, with *F* set to zero for negative *F*^2^. The threshold expression of *F*^2^ > σ(*F*^2^) is used only for calculating *R*-factors(gt) *etc*. and is not relevant to the choice of reflections for refinement. *R*-factors based on *F*^2^ are statistically about twice as large as those based on *F*, and *R*- factors based on ALL data will be even larger.

### Fractional atomic coordinates and isotropic or equivalent isotropic displacement parameters (Å^2^)

**Table:**

	*x*	*y*	*z*	*U*_iso_*/*U*_eq_	
Na1	0.00796 (4)	−0.05352 (15)	0.63208 (5)	0.0255 (2)	
P1	0.19558 (3)	−0.34064 (9)	0.67298 (3)	0.01895 (15)	
N1	0.25953 (9)	−0.1072 (3)	0.70535 (11)	0.0226 (4)	
H1A	0.2462	0.0412	0.6824	0.027*	
O1	0.13141 (7)	−0.2293 (3)	0.59817 (9)	0.0262 (3)	
O2	0.17345 (7)	−0.4754 (3)	0.75752 (9)	0.0258 (3)	
O3	0.24236 (7)	−0.5523 (2)	0.62311 (9)	0.0229 (3)	
O4	−0.10232 (8)	0.2335 (3)	0.60562 (12)	0.0277 (3)	
H1	−0.1189 (14)	0.225 (5)	0.548 (2)	0.043 (7)*	
H2	−0.1341 (14)	0.175 (5)	0.6360 (18)	0.037 (7)*	
C1	0.33360 (10)	−0.1273 (4)	0.76672 (13)	0.0202 (4)	
C2	0.38995 (11)	0.0585 (4)	0.76176 (14)	0.0264 (4)	
H2A	0.3788	0.1914	0.7176	0.032*	
C3	0.46266 (11)	0.0463 (4)	0.82238 (15)	0.0307 (5)	
H3A	0.5000	0.1712	0.8186	0.037*	
C4	0.48004 (11)	−0.1499 (4)	0.88834 (14)	0.0280 (5)	
H4A	0.5286	−0.1570	0.9293	0.034*	
C5	0.42426 (12)	−0.3354 (4)	0.89252 (14)	0.0303 (5)	
H5A	0.4357	−0.4687	0.9364	0.036*	
C6	0.35148 (11)	−0.3260 (4)	0.83237 (14)	0.0269 (5)	
H6A	0.3146	−0.4526	0.8359	0.032*	
C7	0.28512 (11)	−0.4788 (4)	0.54770 (14)	0.0260 (5)	
H7A	0.3373	−0.4213	0.5765	0.031*	
H7B	0.2582	−0.3404	0.5098	0.031*	
C8	0.29054 (15)	−0.7019 (4)	0.48421 (16)	0.0400 (6)	
H8A	0.3188	−0.6552	0.4339	0.060*	
H8B	0.2387	−0.7570	0.4556	0.060*	
H8C	0.3176	−0.8376	0.5221	0.060*	
O5	0.06084 (9)	0.2486 (3)	0.52189 (10)	0.0300 (4)	
H3	0.104 (2)	0.183 (6)	0.539 (2)	0.075 (11)*	
H4	0.0714 (17)	0.400 (7)	0.532 (2)	0.074 (11)*	
O6	−0.06148 (9)	−0.3372 (3)	0.72157 (10)	0.0260 (3)	
H5	−0.0739 (13)	−0.449 (5)	0.6832 (18)	0.041 (8)*	
H6	−0.0987 (17)	−0.250 (6)	0.732 (2)	0.056 (9)*	

### Atomic displacement parameters (Å^2^)

**Table:**

	*U*^11^	*U*^22^	*U*^33^	*U*^12^	*U*^13^	*U*^23^
Na1	0.0261 (4)	0.0264 (4)	0.0236 (4)	−0.0001 (3)	0.0032 (3)	−0.0005 (3)
P1	0.0184 (3)	0.0209 (3)	0.0177 (3)	0.0000 (2)	0.00362 (18)	−0.0014 (2)
N1	0.0232 (8)	0.0174 (9)	0.0252 (8)	0.0021 (7)	−0.0008 (6)	0.0025 (7)
O1	0.0208 (7)	0.0350 (8)	0.0218 (7)	0.0044 (6)	0.0006 (5)	−0.0008 (6)
O2	0.0258 (7)	0.0310 (8)	0.0222 (7)	−0.0046 (6)	0.0083 (5)	−0.0009 (6)
O3	0.0272 (7)	0.0195 (7)	0.0243 (7)	0.0003 (6)	0.0107 (5)	0.0008 (5)
O4	0.0291 (8)	0.0325 (9)	0.0218 (8)	−0.0017 (7)	0.0055 (7)	0.0019 (6)
C1	0.0211 (9)	0.0216 (10)	0.0178 (9)	0.0005 (8)	0.0028 (7)	−0.0037 (8)
C2	0.0295 (11)	0.0220 (10)	0.0276 (10)	0.0001 (9)	0.0049 (8)	0.0046 (8)
C3	0.0232 (10)	0.0331 (12)	0.0356 (12)	−0.0085 (9)	0.0049 (9)	−0.0049 (10)
C4	0.0225 (10)	0.0328 (12)	0.0265 (10)	−0.0003 (9)	−0.0013 (8)	−0.0026 (9)
C5	0.0325 (11)	0.0303 (12)	0.0252 (10)	0.0026 (10)	−0.0026 (8)	0.0068 (9)
C6	0.0270 (10)	0.0236 (11)	0.0285 (10)	−0.0039 (9)	0.0007 (8)	0.0043 (9)
C7	0.0278 (10)	0.0267 (11)	0.0263 (10)	0.0028 (9)	0.0123 (8)	0.0033 (8)
C8	0.0581 (15)	0.0351 (14)	0.0317 (12)	−0.0030 (11)	0.0213 (10)	−0.0043 (10)
O5	0.0309 (9)	0.0279 (9)	0.0296 (8)	−0.0045 (8)	0.0013 (6)	−0.0003 (7)
O6	0.0291 (8)	0.0238 (8)	0.0250 (8)	0.0021 (7)	0.0044 (6)	−0.0015 (7)

### Geometric parameters (Å, °)

**Table:**

Na1—O6^i^	2.3818 (16)	C2—C3	1.388 (3)
Na1—O6	2.4061 (16)	C2—H2A	0.9300
Na1—O4	2.4113 (16)	C3—C4	1.382 (3)
Na1—O1	2.4543 (15)	C3—H3A	0.9300
Na1—O5^ii^	2.4902 (17)	C4—C5	1.382 (3)
Na1—O5	2.5014 (18)	C4—H4A	0.9300
Na1—Na1^ii^	3.7040 (16)	C5—C6	1.386 (3)
Na1—H3	2.61 (3)	C5—H5A	0.9300
P1—O2	1.4885 (14)	C6—H6A	0.9300
P1—O1	1.5030 (14)	C7—C8	1.485 (3)
P1—O3	1.6089 (14)	C7—H7A	0.9700
P1—N1	1.6612 (16)	C7—H7B	0.9700
N1—C1	1.415 (2)	C8—H8A	0.9600
N1—H1A	0.8600	C8—H8B	0.9600
O3—C7	1.448 (2)	C8—H8C	0.9600
O4—H1	0.81 (3)	O5—H3	0.82 (3)
O4—H2	0.81 (3)	O5—H4	0.83 (3)
C1—C6	1.390 (3)	O6—H5	0.80 (3)
C1—C2	1.392 (3)	O6—H6	0.83 (3)
			
O6^i^—Na1—O6	90.09 (4)	C6—C1—C2	119.07 (17)
O6^i^—Na1—O4	90.48 (6)	C6—C1—N1	121.88 (17)
O6—Na1—O4	90.65 (6)	C2—C1—N1	119.04 (17)
O6^i^—Na1—O1	97.38 (6)	C3—C2—C1	120.32 (19)
O6—Na1—O1	113.74 (6)	C3—C2—H2A	119.8
O4—Na1—O1	154.22 (6)	C1—C2—H2A	119.8
O6^i^—Na1—O5^ii^	173.68 (7)	C4—C3—C2	120.5 (2)
O6—Na1—O5^ii^	89.39 (6)	C4—C3—H3A	119.7
O4—Na1—O5^ii^	83.23 (7)	C2—C3—H3A	119.7
O6^i^—Na1—O5	95.66 (6)	C3—C4—C5	119.05 (17)
O6—Na1—O5	171.35 (6)	C3—C4—H4A	120.5
O4—Na1—O5	82.86 (7)	C5—C4—H4A	120.5
O1—Na1—O5	71.99 (6)	C4—C5—C6	121.06 (19)
O5^ii^—Na1—O5	84.19 (6)	C4—C5—H5A	119.5
O6—Na1—Na1^ii^	131.34 (5)	C6—C5—H5A	119.5
O4—Na1—Na1^ii^	80.61 (5)	C5—C6—C1	119.95 (19)
O1—Na1—Na1^ii^	76.97 (5)	C5—C6—H6A	120.0
O5—Na1—Na1^ii^	41.98 (4)	C1—C6—H6A	120.0
O6^i^—Na1—H3	90.9 (7)	O3—C7—C8	108.78 (16)
O6—Na1—H3	168.7 (8)	O3—C7—H7A	109.9
O4—Na1—H3	100.6 (8)	C8—C7—H7A	109.9
O1—Na1—H3	54.9 (8)	O3—C7—H7B	109.9
O5^ii^—Na1—H3	90.8 (7)	C8—C7—H7B	109.9
O5—Na1—H3	18.4 (8)	H7A—C7—H7B	108.3
Na1^ii^—Na1—H3	50.8 (7)	C7—C8—H8A	109.5
O2—P1—O1	118.43 (8)	C7—C8—H8B	109.5
O2—P1—O3	103.77 (8)	H8A—C8—H8B	109.5
O1—P1—O3	109.53 (7)	C7—C8—H8C	109.5
O2—P1—N1	112.93 (8)	H8A—C8—H8C	109.5
O1—P1—N1	106.02 (8)	H8B—C8—H8C	109.5
O3—P1—N1	105.43 (8)	Na1^ii^—O5—Na1	95.81 (6)
C1—N1—P1	126.77 (13)	Na1^ii^—O5—H3	111 (2)
C1—N1—H1A	116.6	Na1—O5—H3	88 (2)
P1—N1—H1A	116.6	Na1—O5—H4	127 (2)
P1—O1—Na1	124.98 (7)	H3—O5—H4	101 (3)
C7—O3—P1	119.75 (12)	Na1—O6—H5	101.8 (17)
Na1—O4—H1	104.5 (18)	Na1—O6—H6	103 (2)
Na1—O4—H2	106.0 (17)	H5—O6—H6	114 (3)
H1—O4—H2	110 (2)		

Symmetry codes: (i) −*x*, *y*+1/2, −*z*+3/2; (ii) −*x*, −*y*, −*z*+1.

### Hydrogen-bond geometry (Å, °)

**Table:**

*D*—H···*A*	*D*—H	H···*A*	*D*···*A*	*D*—H···*A*
N1—H1A···O3^iii^	0.86	2.29	3.132 (2)	166
O4—H1···O1^ii^	0.81 (3)	2.02 (3)	2.808 (2)	165 (2)
O4—H2···O2^i^	0.82 (2)	1.92 (3)	2.693 (2)	159 (2)
O5—H4···O1^iii^	0.82 (4)	2.32 (3)	3.116 (2)	162 (3)
O6—H5···O4^iv^	0.80 (3)	2.01 (3)	2.797 (2)	171 (3)
O6—H6···O2^i^	0.83 (3)	1.96 (3)	2.771 (2)	166 (3)

Symmetry codes: (iii) *x*, *y*+1, *z*; (ii) −*x*, −*y*, −*z*+1; (i) −*x*, *y*+1/2, −*z*+3/2; (iv) *x*, *y*−1, *z*.
